# The Characteristics of Antioxidant Activity after Liver Transplantation in Biliary Atresia Patients

**DOI:** 10.1155/2015/421413

**Published:** 2015-05-12

**Authors:** Chih-Jen Chen, Kuo-Shu Tang, Ying-Hsien Huang, Chao-Long Chen, Li-Tung Huang, Jiin-Haur Chuang, Mao-Meng Tiao

**Affiliations:** ^1^Department of Pediatrics, Kaohsiung Chang Gung Memorial Hospital and Chang Gung University College of Medicine, Kaohsiung 83301, Taiwan; ^2^Department of Surgery, Kaohsiung Chang Gung Memorial Hospital and Chang Gung University College of Medicine, Kaohsiung 83301, Taiwan

## Abstract

*Purpose*. Cholestatic liver injury is associated with a high production of free radicals. The pathogenesis of liver injury in biliary atresia (BA) patients is largely undefined. The goal of the present study was to clarify the oxidative damage and the changes in antioxidant enzyme activities that occur during the development of BA and after liver transplantation (LT). *Methods*. We enrolled BA patients and control subjects and collected their clinical information. The activities of antioxidant enzymes in BA patients before LT (BA group) and after LT (LT group) were analyzed. *Results*. The number of mitochondrial DNA copies had increased in the LT group compared with the BA group. Similarly, the activity of glutathione peroxidase had increased in the LT group compared with the BA group. The level of glutathione was higher in the LT group than in the BA group. Malondialdehyde levels were decreased in the LT group compared with the BA group. *Conclusions*. These data indicate that LT is associated with increased antioxidant enzyme activities and decreased malondialdehyde levels in BA patients. The manipulation of mitochondria-associated antioxidative activity might be an important future management strategy for BA.

## 1. Introduction

Biliary atresia (BA) is an important clinical problem involving impairments in bile flow that manifest as neonatal jaundice and can lead to progressive fibrosis and end-stage liver cirrhosis [1, 2]. Oxidative stress and mitochondrial dysfunction are involved in the pathogenesis of chronic liver cholestasis [3]. Oxidative stress in cells or tissues is an abnormal phenomenon that occurs when the production of oxygen radicals exceeds that of the antioxidant capacity [4]. These excess free radicals damage essential cellular macromolecules, leading to abnormal gene expression and cell death [4]. Antioxidant enzymes catalyze the decomposition of these free radicals. The major antioxidant enzymes include glutathione peroxidase (GPx) and catalase (CAT), which differ in structure, tissue distribution, and cofactor requirements [5].

Disturbances in the antioxidant system could play a role in the pathogenesis of chronic liver disease [6–8] and could result in different types of liver diseases in infancy [9]. It is reported that serum oxidative stress indexes were significantly higher in the inherited metabolic diseases group than in the biliary atresia group [10]. GPx is the most important scavenger of hydrogen peroxide (H_2_O_2_) in mammalian cells [11]. CAT, which is located in peroxisomes of all aerobic cells [12], is an essential enzyme in the decomposition of intracellular H_2_O_2_, promoting the breakdown of H_2_O_2_ into water and oxygen without the production of free radicals.

The copy numbers of mitochondrial DNA (mtDNA) in cells may change during cell growth and differentiation [13]. mtDNA is highly susceptible to oxidative stress that leads to mitochondrial dysfunction [3, 14]. Previous studies suggest that an increase in the mtDNA copy number in cells is the result of a feedback mechanism that compensates for defective mitochondria that affect subsequent cell growth and morphology [14, 15]. Extrahepatic cholestatic patients present a significant decrease in the number of mtDNA copies compared with controls [3].

Our previous report demonstrated that the level of oxidative stress is lower after the Kasai operation. In addition, the mtDNA copy number is found to be higher after the Kasai operation [2]. To our knowledge, no study has evaluated the benefit of liver transplantation (LT) in BA patients in terms of oxidants/antioxidants. Our hypothesis is that if antioxidant levels are increased after LT [2, 10, 16, 17], then the administration of antioxidants is needed in those cases who hesitate to receive LT or in cases where a liver graft is not available from donors. Therefore, our aim was to study the oxidative status in children with BA by examining the malondialdehyde (MDA) and antioxidant enzyme levels as well as mitochondrial copy numbers.

## 2. Materials and Methods

### 2.1. Subjects

Participants were enrolled from the clinic at the Chang Gung Memorial Hospital, Department of Pediatrics, and liver team at the Kaohsiung Medical Center. In all patients, BA was diagnosed clinically with DISIDA scan, pathologic examination, computerized tomography, or magnetic resonance imaging findings before LT. The indications for LT among BA patients included end-stage liver disease, recurrent cholangitis, recurrent gastrointestinal bleeding, and portal hypertension. Patients who received only cyclosporine after LT were included. Those who received other immunosuppressants were excluded. Normal healthy individuals who tested negative in the allergy screen test were included as a control group. Another control group consisted of patients with idiopathic acute hepatitis. The liver tissues were randomly obtained during Kasai operation. Tissue samples from hepatitis cases were randomly obtained using a modified Minghini needle (sure cut), and biliary atresia cases who received LT had tissue samples randomly obtained at 6 months after LT. Liver tissues of normal controls were obtained from the LT donors. They were stored at −80°C till assay. The total bilirubin levels and alanine transaminase (ALT) and aspartate transaminase (AST) activities were determined by a standard autoanalyzer (model 7450; Hitachi, Tokyo, Japan). The study was approved by the Ethics and Clinical Research Committee of the Chang Gung Memorial Hospital.

### 2.2. GPx Activity and Glutathione

GPx activity was measured using a commercially available kit (Ransel; Randox Lab, Crumlin, UK) for erythrocytes and homogenized liver tissue. Liver biopsy samples were washed in phosphate buffer at pH 7.4. Then, the tissue was homogenized in 5 mL/g cold buffer, which consisted of 50 mM Tris-HCl, pH 7.5, 5 mM EDTA, and 1 nM dithiothreitol. The homogenate was centrifuged at 10,000 g for 15 minutes at 4°C. The supernatant was removed for assay of GPx. In the presence of glutathione reductase (GR) and NADPH, oxidized glutathione (GSSG) was immediately converted to the reduced form (GSH) with a concomitant oxidation of NADPH to NADP^+^. The decrease in absorbance at 340 nm after 1 and 2 min was measured. The result obtained was expressed in units per liter (U/L) of hemolysate and was multiplied by the appropriate dilution factor to obtain the result in U/L.

### 2.3. Determination of CAT Activity

The plasma and liver supernatant assays were conducted on a Bradford assay (Bio-Rad, Richmond, CA, USA) automated chemistry analyzer. The CAT activity assay kit (Cayman Chemical, Ann Arbor, MI, USA; catalog number 707002) and samples (20 *μ*L) were placed on the instrument and assayed according to the manufacturer's protocol. Liver biopsy was washed with phosphate buffer, pH 7.4, to remove red blood cells. The tissue was then blotted dry, weighted, and followed by homogenization in 1.5 mL cold buffer (50 mM potassium phosphate and 1 mM EDTA, pH 7) and centrifugation at 10,000 g for 15 minutes at 4°C. The supernatant was used for the assay. The CAT activity level was quantified spectrophotometrically at 540 nm. The results were expressed as nanomoles per minute per milliliter of protein. Standards and blanks were assayed in duplicate and treated as samples (i.e., not placed in the calibration positions). The average of 2 measurements was used in subsequent statistical analysis of the data.

### 2.4. Determination of Plasma MDA Content

MDA is an end product of peroxidative decomposition of polyenoic fatty acids in the lipid peroxidation process, and its accumulation in tissues is indicative of the extent of lipid peroxidation. Plasma MDA was measured using the thiobarbituric acid reactive substances (TBARS) assay. The TBARS reagent (1 mL) was added to an aliquot containing 0.5 mL plasma, and the mixture was heated for 20 min at 100°C. The antioxidant, butylated hydroxytoluene, was added before heating the samples. After cooling on ice, samples were centrifuged at 840 g for 15 minutes, and the absorbance of the supernatant was read at 532 nm (A532). Blanks for each sample were prepared and assessed in the same way to correct for the contribution of A532 to the sample. TBARS results were expressed as MDA equivalents by using 1,1,3,3-tetraethoxypropane as the standard.

### 2.5. Determination of mtDNA Copy Number

DNA samples were extracted from peripheral blood leukocytes. The mtDNA copy numbers were measured by real-time polymerase chain reaction (PCR) after correcting nuclear DNA levels. The forward and reverse primers complementary to nuclear *β-actin* gene were 5′-GAAATCGTGCGTGACATTAAAG-3′ and 5′-ATCGGAACCGCTCATTG-3′. The forward and reverse primers for mtDNA, which were complementary to the sequence of the mitochondrial* ND1* gene, were 5′-ATTCTAGCCACATCAAGTCTTT-3′ and 5′-GGAGGACGGATAAGAGGATAAT-3′. PCR was performed in a LightCycler 480 Real-Time PCR System (Roche Co., Mannheim, Germany), using LightCycler 480 SYBR Green I Master (Roche Co., Germany). DNA (10 ng) was mixed with 10 *μ*L SYBR Green PCR Master Mix containing 10 nmol of forward and reverse primers, in a final volume of 20 *μ*L. The PCR conditions were as follows: initial 50°C 2 min, 95°C 1 min, 40 cycles of denaturation at 95°C for 15 s, annealing at 60°C for 20 s, and primer extension at 72°C for 15 s, final 25°C. The threshold cycle number (Ct) values of the *β-actin* gene and the mitochondrial* ND1* gene were determined for each individual quantitative PCR run. Ct values were used as a measure of the input copy number, and Ct value differences were used to quantify mtDNA copy number relative to the *β-actin* gene according to the following equation: relative copy number (Rc) = 2^ΔCt^, where ΔCt is Ct_*β-actin*_ − Ct_mtDNA_ [18]. Each measurement was performed at least 3 times and was normalized in each experiment against a serial dilution of a control DNA sample.

### 2.6. Statistical Analysis

SPSS for Windows version 13.0 (SPSS Inc., Chicago, IL, USA) was used for statistical analysis. Continuous variables were analyzed by independent* t*-test or ANOVA. The correlation of total bilirubin levels and oxidative stress and antioxidant enzyme activity was performed using Spearman's correlation analysis. Data are presented as means ± SE. *P* < 0.05 was considered statistically significant. The sample size was calculated with PASS sample size software, the two-sided confidence level >0.95, *P* < 0.05, and power value >0.8 with *α* level <0.05.

## 3. Results

### 3.1. Patients' Clinical Data

This study included 26 normal healthy controls, 26 post-Kasai operation BA patients before and after LT, and 6 acute hepatitis patients. The descriptive data of the children included in this study are shown in Table 1. The liver function and levels of AST, ALT, and bilirubin were increased in the BA and acute hepatitis groups compared with the normal control group and significantly decreased in the LT group compared with the BA group (Table 1).

### 3.2. Oxidative Stress and Antioxidant Enzyme Activity

**Figure 1:**
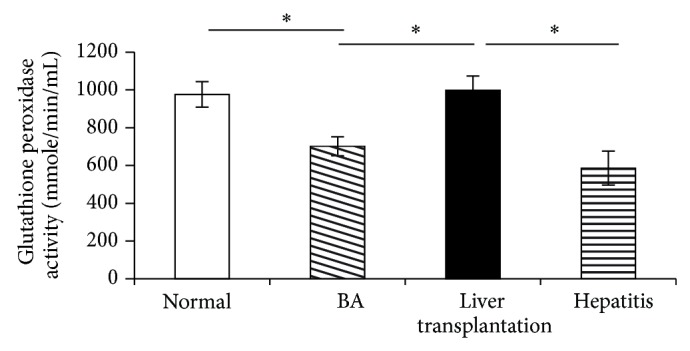
The activity of the antioxidant glutathione peroxidase in plasma was significantly decreased in the biliary atresia group (BA) before liver transplantation (LT) compared with the normal control group and significantly increased after undergoing liver transplantation in the LT group compared with the BA group, almost reaching the level of the normal control group. Data represent the mean ± SE. ^∗^ *P* < 0.05 versus BA.

The activity of the antioxidant GPx significantly increased in the LT group compared with the BA group, almost reaching the level in the normal control group and significantly decreased in the BA group compared with the normal controls (Figure 1).

**Figure 2:**
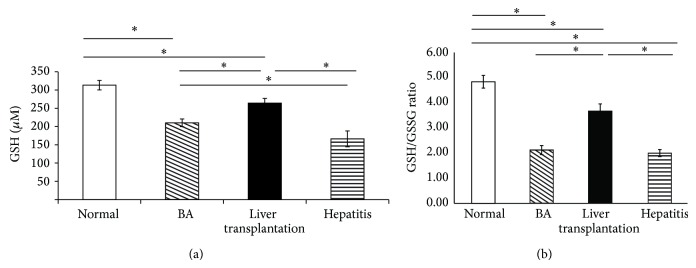
The expression of (a) total glutathione (GSH) and (b) the GSH/glutathione disulfide (GSSG) ratio in plasma significantly decreased in the biliary atresia group (BA) before liver transplantation (LT) compared with the normal control group and significantly increased after undergoing liver transplantation in the LT group compared with the BA group, almost to the level of the normal control group. Data represent the mean ± SE. ^∗^ *P* < 0.05 versus BA.

The expression of glutathione (GSH) is significantly increased in the LT group compared with the BA group, almost reaching the level in the normal control group and significantly decreased in the BA group compared with the normal control group (Figure 2(a)). The GSH/glutathione disulfide ratio significantly increased in the LT group compared with the BA group (Figure 2(b)).

**Figure 3:**
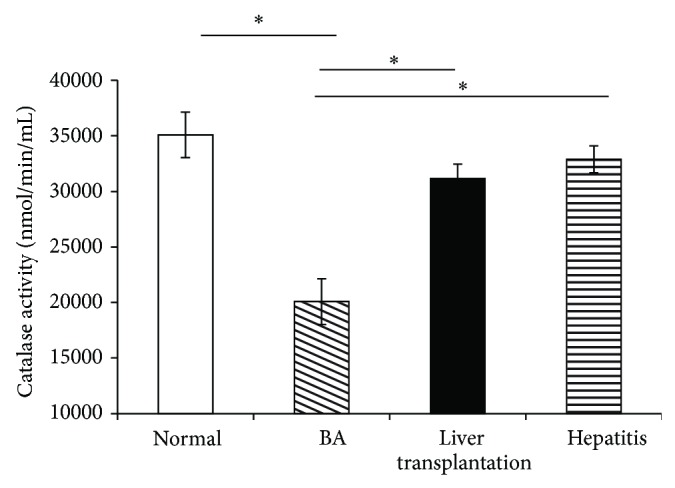
The expression of catalase in plasma is significantly decreased in the biliary atresia (BA) group before liver transplantation (LT) compared with the normal control group and increased after undergoing LT. Data represent the mean ± SE. ^∗^ *P* < 0.05 versus BA.

The expression of CAT significantly increased in the LT group compared with the BA group and decreased in the BA group compared with the normal control group (Figure 3, Table 2).

**Figure 4:**
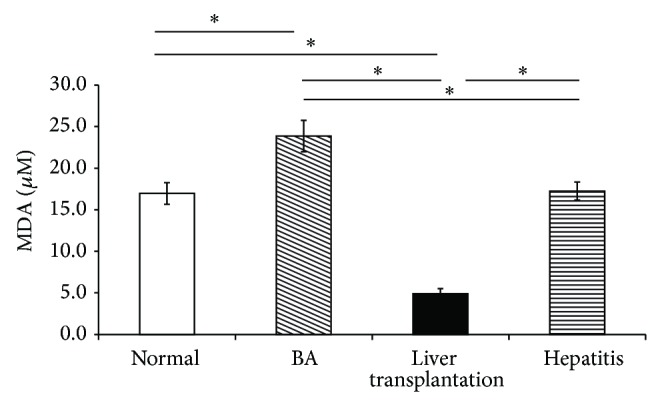
The malondialdehyde (MDA) level in plasma decreased in patients after liver transplantation (LT) compared with biliary atresia (BA) patients before LT and the hepatitis group. Data represent the mean ± SE. ^∗^ *P* < 0.05 versus BA.

The MDA level decreased in the LT group compared with the BA and hepatitis groups (Figure 4).

### 3.3. Correlations between the Severity of Cholestasis and Oxidative Stress and Antioxidant Enzyme Activity

The severity of cholestasis is directly related to the oxidative stress level and inversely related to the antioxidative level [19]. The total bilirubin level significantly correlated with the levels of MDA (*r* = 0.507, *P* < 0.001), GPx (*r* = −0.440, *P* = 0.002), and GSH (*r* = −0.478, *P* = 0.001). The total bilirubin level significantly correlated with the levels of AST (*r* = 0.612, *P* < 0.001) and ALT (*r* = 0.467, *P* = 0.001). However, the correlation between total bilirubin and CAT levels (*r* = −0.022, *P* = 0.887) and mtDNA copy number (*r* = 0.006, *P* = 0.971) was not significant.

### 3.4. Post-LT mtDNA Copy Numbers

**Figure 5:**
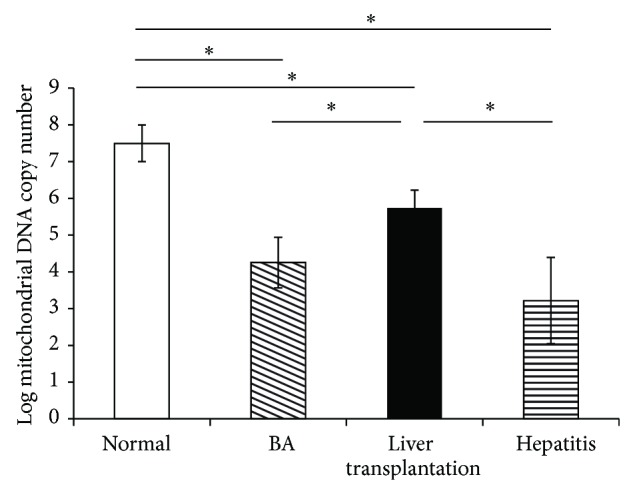
Comparison of the mitochondrial DNA (mtDNA) copy number in plasma between patients with biliary atresia (BA) and those receiving liver transplantation (LT), using the relative copy number (Rc) = 2^ΔCt^, where ΔCt is Ct_*β-actin*_ − Ct_mtDNA_. The mtDNA copy number increased in the LT group compared with the BA group. Data represent the mean ± SE. ^∗^ *P* < 0.05 versus BA.

The mtDNA copy numbers were compared between the BA and LT groups by calculating the relative copy number (Rc) = 2^ΔCt^, where ΔCt is Ct_*β-actin*_ − Ct_mtDNA_. The mtDNA copy numbers increased in the LT group compared with the BA group (Figure 5).

## 4. Discussion

This study more clearly indicates that both the mitochondrial number and the level of oxidative stress are involved in BA and that both the number of mitochondria and their antioxidant enzymes or their activity recover after LT. Therefore, the manipulation of mitochondria-associated antioxidative activity may be important in the management of BA even after LT. A thorough understanding of the mitochondrial influence on disease occurrence and progression is therefore crucial for the treatment of BA patients.

BA-induced obstructive jaundice in children is an important clinical issue [1, 20, 21] that is associated with high morbidity and mortality rates [1]. Bile flow impairment leads to progressive liver injury, fibrosis, and end-stage liver cirrhosis. If not treated properly and effectively, LT is required for survival in most cases [1]. The pathogenesis of this disease is largely undefined, and there are no effective strategies to prevent its development. Studies exploring the underlying mechanisms, in particular those investigating protein profiles, have been limited by the inability to investigate the biliary tree at the onset of disease and to trace the progression of bile duct obstruction at defined phases. Many studies have reported increased antioxidant enzyme levels in BA and other liver diseases [9, 22, 23], although some demonstrated a decrease in antioxidant enzyme levels [7, 8]. In this study, we evaluated the levels of several antioxidant enzymes and oxidative stress markers at different stages, with emphasis on the BA and post-LT conditions, and gained a clearer understanding of BA pathogenesis that is relevant to its management strategy.

Oxidative stress is a major pathogenic event that occurs in several liver disorders, ranging from metabolic to proliferative conditions, and is a major cause of liver damage during LT [24]. Numerous studies have shown that oxidative stress is involved in the pathogenesis of cholestasis [25] and lipid peroxidation is responsible for the tissue injury that occurs in this condition [26]. End-stage liver cirrhosis accounts for approximately one-third of patients referred for LT [25]. Several enzymatic (i.e., CAT and GPx) and nonenzymatic (i.e., GSH and MDA levels) markers of chronic oxidative stress in the liver are well known [24]. Patients with jaundice have significantly higher mean levels of plasma lipid peroxides such as MDA and bilirubin than patients without jaundice [27]. Impairment of bile flow is likely to result in the accumulation of toxic hydrophobic bile salts within the hepatocytes, interfering with electron transport with consequent H_2_O_2_ and superoxide (O_2_^−^) formation [9, 22, 28]. Identification of these markers will enable the early detection of liver diseases and will allow for monitoring the degree of liver damage, response to pharmacological therapies, and development of new therapeutic approaches [24, 29]. Many studies in humans and in rat models have been conducted to clarify the relationship between oxidative stress and antioxidants in the liver. In humans, an increase in the production of free oxygen radicals activates a complex defense system. This system includes GPx, CAT, GSH, glutathione reductase, and vitamins [26]. Our study showed a significant decrease in GSH, GPx, and CAT activities in patients with BA compared with their activities in the control group, which recovered after LT. In line with Salem et al.'s study [7], the levels of CAT in hepatitis or liver cirrhosis were low and presumably related to the effects of oxidative stress [8]. A marked decrease in the antioxidant status may lead to excess oxygen free radical formation, which promotes pathological processes in the liver.

In Granot et al.'s study [29], the levels of oxidants and antioxidants did not differ between cyclosporine A- (CsA-) treated patients after LT and healthy controls. In our study, markedly higher oxidative stress levels were related to more severe cholestasis. CsA binds to lipoproteins, primarily low-density lipoprotein phospholipids, which are expected to be particularly susceptible to lipid peroxidation [29]. In this study, we determine whether the high antioxidative enzyme levels were induced by LT or by CsA. More research is needed to clarify this issue. The administration of suitable antioxidant therapy just before the reestablishment of blood flow to the graft is important to prevent plasma redox imbalance and to prevent or ameliorate post-LT cholestatic injury of the liver graft [30].

Mitochondria are the major supplier of energy in mammalian cells [31] and provide energy necessary for the damage repair and cellular survival in response to the exposure of the environment during disease processes such as oxidative stress [31]. To meet this energy demand, signals are transmitted to the nucleus for inducing mitochondrial proliferation and mtDNA amplification for the production of a high number of functional mitochondria. In this study, the effect of mitochondria-associated oxidative stress on the development of BA that recovered after LT was observed by examining the plasma redox state and the mtDNA mass. On the basis of our previous studies, oxidative stress and mitochondrial intrinsic apoptosis pathways are involved in liver cell death in the bile duct ligation-induced cholestatic rat model [20, 32, 33]. Early-stage BA is associated with augmented oxidative DNA, mtDNA damage, and a decrease in mitochondrial copy number relative to that observed in late-stage BA. Our study was in accordance with Asakawa's which described that postoperative patients with BA were under increased oxidative stress [17]. Our report also proved that the oxidative stress increased more in BA before Kasai operation than after the operation and both are higher than the normal control [2]. Antioxidant therapy might be necessary to decrease oxidative stress in postoperative patients with BA [17]. Thus, altered mitochondrial function and increase in mitochondrial numbers after LT, in BA pathogenesis, will provide detailed information about the effects of oxidative stress on the development of BA, which improves after LT. These findings also suggest that mitochondrial and oxidative stress variations could serve as useful biomarkers for early BA treatment in the future.

In conclusion, the current study demonstrates that GPx, CAT, and GSH levels are low in patients with BA cholestasis. The significant increase in their levels after LT may point to their role as key enzymes in the protection of the liver from the hazards of free radical reactions and may reflect the appropriate activity of antioxidant barrier enzymes as a response to increased oxidative stress. Increased knowledge of redox regulation may have important clinical ramifications for understanding the pathogenesis of BA liver diseases and for developing therapeutic approaches.

## Figures and Tables

**Table 1:** Descriptive data of the children.

Groups	Normal	BA	LT	Hepatitis
	(*n* = 26)	(*n* = 26)	(*n* = 26)	(*n* = 6)
Age (yr) (mean ± standard error [SE])	2.1 ± 1.5	1.9 ± 0.8	8.6 ± 0.9	2.4 ± 1.2
Sex (male/female)	1.1 : 1	1.1 : 1	0.8 : 1	0.8 : 1
Physical signs, number (%)				
Jaundice	0 (0)	26 (100)	0 (0)	6 (100)
Edema of the lower limbs	0 (0)	0 (0)	0 (0)	0 (0)
Hepatomegaly	0 (0)	8 (30.7)	3 (11.5)	2 (33.3)
Splenomegaly	0 (0)	11 (42.3)	3 (11.5)	1 (16.7)
Ascites	0 (0)	2 (7.6)	0 (0)	0 (0)
Liver function tests (mean ± SE)				
bil(D) (mg/dL)	0.2 ± 0.1^**∗∗**^	4.4 ± 0.8^**∗**^	0.3 ± 0.1^**∗∗**^	3.4 ± 0.9^**∗**^
bil(T) (mg/dL)	0.50 ± 0.2^**∗∗**^	6.5 ± 1.2^**∗**^	0.8 ± 0.1^**∗∗**^	5.1 ± 1.3^**∗**^
AST (U/L)	35.8 ± 3.8^**∗∗**^	163.4 ± 30.5^**∗**^	32.7 ± 5.7^**∗∗**^	341.9 ± 177.3^**∗**^
ALT (U/L)	21.4 ± 5.6^**∗∗**^	121.8 ± 26.4^**∗**^	36.5 ± 6.4^**∗∗**^	184.0 ± 110.9^**∗**^
ALP (U/L)	150.3 ± 15.4^**∗∗**^	510.0 ± 81.3^**∗**^	288.5 ± 25.8^**∗∗**^	314.0 ± 152.0^**∗**^
Albumin (g [%])	4.1 ± 0.2	3.0 ± 0.6	4.0 ± 0.4	3.1 ± 0.8

BA: biliary atresia before liver transplantation; LT: biliary atresia after liver transplantation; bil(D): direct bilirubin; bil(T): total bilirubin; AST: aspartate aminotransferase; ALT: alanine aminotransferase; ALP: alkaline phosphatase. All results represent means ± standard error (SE). ^**∗**^ *P* < 0.05 versus normal; ^**∗∗**^ *P* < 0.05 versus BA.

**Table 2:** Comparison of liver supernatant (randomly selected cases) between mean levels (±SE) of glutathione peroxidase and catalase (CAT) enzymes in different cholestasis groups and control children.

Variable	GPx (nmol/min/mL)	CAT (nmol/min/mL)
Normal (*n* = 3)	67.01 ± 6.32^**∗**^	10.57 ± 2.37^**∗**^
BA (*n* = 10)	50.05 ± 4.26	7.11 ± 1.53
LT (*n* = 10)	72.48 ± 5.21^**∗**^	12.00 ± 1.34^**∗**^
Hepatitis (*n* = 4)	49.01 ± 3.34	10.26 ± 1.62

BA: biliary atresia before liver transplantation; CAT: catalase; GPx: glutathione peroxidase; LT: biliary atresia after liver transplantation;  ^**∗**^ *P* < 0.05 versus BA.

## Abbreviations

BA: Biliary atresia
CAT: Catalase
GPx: Glutathione peroxidase
H_2_O_2_: Hydrogen peroxide
LT: Liver transplantation
MDA: Malondialdehyde
mtDNA: Mitochondrial DNA
ROS: Reactive oxygen species.
